# Kidnapping intergroup young: an alternative strategy to maintain group size in the group-living pied babbler (*Turdoides bicolor*)

**DOI:** 10.1098/rstb.2021.0153

**Published:** 2022-05-23

**Authors:** Amanda R. Ridley, Martha J. Nelson-Flower, Elizabeth M. Wiley, David J. Humphries, Hanna Kokko

**Affiliations:** ^1^ Centre for Evolutionary Biology, School of Biological Sciences, University of Western Australia, Perth, Australia; ^2^ Percy FitzPatrick Institute of African Ornithology, University of Cape Town, Cape Town, Western Cape, South Africa; ^3^ Department of Biology, Langara College, Vancouver, BC, Canada; ^4^ Institute of Evolutionary Biology and Environmental Studies, University of Zurich, Zurich, Switzerland

**Keywords:** cooperation, kidnapping, intergroup interactions, extinction, pied babbler, *Turdoides bicolor*

## Abstract

Both inter- and intragroup interactions can be important influences on behaviour, yet to date most research focuses on intragroup interactions. Here, we describe a hitherto relatively unknown behaviour that results from intergroup interaction in the cooperative breeding pied babbler: kidnapping. Kidnapping can result in the permanent removal of young from their natal group. Since raising young requires energetic investment and abductees are usually unrelated to their kidnappers, there appears no apparent evolutionary advantage to kidnapping. However, kidnapping may be beneficial in species where group size is a critically limiting factor (e.g. for reproductive success or territory defence). We found kidnapping was a highly predictable event in pied babblers: primarily groups that fail to raise their own young kidnap the young of others, and we show this to be the theoretical expectation in a model that predicts kidnapping to be facultative, only occurring in those cases where an additional group member has sufficient positive impact on group survival to compensate for the increase in reproductive competition. In babblers, groups that failed to raise young were also more likely to accept extragroup adults (hereafter rovers). Groups that fail to breed may either (i) kidnap intergroup young or (ii) accept rovers as an alternative strategy to maintain or increase group size.

This article is part of the theme issue ‘Intergroup conflict across taxa’.

## 1. Introduction

Group-living behaviour is assumed to occur when the benefits of living in a group outweigh the costs. Numerous studies provide convincing evidence of the benefits of group-living behaviour, including increased predator detection [1], increased survival [2–4], load-lightening [2,5], greater success at territorial defence [6,7], stable pair bonds [8] and higher reproductive success [9]. However, the majority of research on group-living focuses on intragroup behaviours, despite the fact that in group-living species groups tend to be surrounded by other groups and may regularly interact with them [6,10,11]. When group-living brings benefits, group size relative to the size of neighbouring groups may become an important determinant of group stability. Unsuccessful breeding can cause a group to decrease in size through lack of recruitment to the adult population, and this is of particular importance if neighbouring groups increase in size relative to the focal group. A small group size relative to surrounding groups can result in greater chance of territory loss [6], breeding interference [12], dominance takeover [13] or group extinction [14,15].

When breeding fails, there can be alternative strategies to increase or maintain group size or defend a territory: a group may make infanticidal attacks on neighbouring groups (reducing the size of neighbouring groups may then yield a relative advantage in territorial disputes) or kidnap individuals from another group. Alternative strategies to increase group size can also involve passive acceptance of new group members, e.g. to form temporally limited flocks of migratory birds [16,17], or longer term group membership when rovers—defined as individuals that leave their original group and actively seek out and approach another group in a non-aggressive manner [18] —are accepted as group members. Here, we focus on a particularly active route to recruiting new group members: raising intergroup young. In this case, a less obvious process known as group augmentation may come into play [19], where current investment in young forms a cost that has to be paid now, while the benefits accrue only later when the young are no longer dependent, and their presence begins to contribute to group-level benefits.

Usually, group augmentation benefits are discussed in the context of alloparenting intragroup young [19]. This creates a combination of indirect and direct (albeit delayed) benefits, making helping behaviour easier to explain. An alternative route to increasing the number of group young is to invest in young produced in other groups. This behaviour is termed ‘kidnapping’, and it is more difficult to explain than intragroup alloparenting, since relatedness-based benefits are unlikely (or at least not guaranteed) to be received from investment in intergroup young.

Data from previous studies suggest that kidnapping could occur for a number of reasons such as (i) a hormonal byproduct behaviour, especially by individuals that have recently lost their own young, or (ii) because the kidnapped individual can bring some benefit to the kidnapper. The case of (i) focuses on finding a proximate cause, and we would not expect the kidnapper to gain any clear benefit from kidnapping. For example, in emperor penguins (*Aptenodytes forsteri*), hundreds of young may be kidnapped in large colonies [20], but kidnapping is temporary (97.7% of kidnappings last only a few hours) and is associated with a hormonal response in individuals that have recently lost their own young [21]. In the case of (ii) however, the kidnapper should gain a clear benefit, as seen in slave-making ants. In this case, kidnapping is well explained: abductees do not require high levels of care before becoming helpers and therefore represent a low-risk investment with a high-return outcome (since abductees become helpers for life). By stealing individuals as pupae, slave-making ants reduce the potential cost associated with kidnapping since pupae do not fight back, and newly emerged slaves will form a social bond with the kidnapper through olfactory imprinting, reducing the likelihood of attempts to return to their natal colonies [22].

In social species, where group size can be an important determinant of fitness, kidnapping intergroup young may bring benefits and indeed kidnapping has been recorded in a number of social species, including Southern ground hornbills *Bucorvus leadbeateri*, [23], naked mole-rats *Heterocephalus glaber* [24], white-winged choughs *Corcorax melanorhamphos* [25], banded mongoose *Mungos mungo* [26] and several primate species [27,28]. Given the increasing frequency at which intergroup kidnapping behaviour is being reported, it is of interest to specify the conditions under which it may be advantageous.

Here, we analyse empirical data on patterns of intergroup kidnapping. We present 16 years of life-history data to investigate patterns of kidnapping behaviour and acceptance of rovers in the cooperatively breeding pied babbler (*Turdoides bicolor*), a group-living species where group size has an important influence on group longevity and reproductive output and territory size can change significantly between years [29–31]. We investigate whether there are consistent patterns that may explain the occurrence of alternative strategies to increase group size (specifically intergroup kidnapping and the acceptance of rovers into the group) and determine whether breeding success may be an important factor driving the occurrence of these behaviours. Based on the results of our empirical data, we present a model that investigates the conditions where group benefits alone are sufficient to counteract the fact that kidnapping brings, genetically speaking, a competitor into the group. The model implies that kidnapping dilutes the probability that future descendants from the group carry the kidnappers' own genes, and we take this cost into account.

## 2. Methods

Life-history data were collected from July 2003 to April 2019 on a population of habituated pied babbler groups in the southern Kalahari (26°58′  S, 20°49′  E). Pied babblers are small–medium cooperatively breeding passerines (65–95 g) that defend a territory year round [30]. The number of social groups in the population range between 9 and 21 annually (average group size 4.04 ± 0.13 adults). Intergroup interactions are common, typically occurring once per 3–4 h observation session, and comprising a vocal and visual display [10,32]. Intergroup interactions in this species can be costly, leading to lower reproductive success [33] and loss of body mass compared to days without intergroup interactions [34], but they can also be a form of information exchange between groups [35].

All adult group members help to raise the young produced by a single breeding pair [36]. Genetic analyses have revealed evidence of inbreeding avoidance in this species [37]. The median brood size is three, ranging between two to five chicks per brood [36]. Young have a prolonged period of post-fledging care and become nutritionally independent (defined as the period where they are mostly foraging for themselves and receive less than one feed per observation hour from adults) at 2–3 months post-fledging [36]. Individuals are defined as adults once they have reached 1 year of age [31]. The breeding season typically spans the summer period from September to April, and up to three different broods may be successfully raised during a single breeding season [36].

The typical lifespan of a wild pied babbler is approximately 6 years, but annual survival is difficult to determine precisely, since missing individuals may have either died or dispersed outside of the study area. Our previous analyses revealed that dispersal distance is the same for both males and females, with no evidence for sex biases in dispersal in this species [37]. The maximum age observed in our population is 14 years, and breeding pair turnover can be low, with some breeding pairs staying bonded for over 5 years [8]. Over the 16 years of observation, some groups were present over many years, while in some cases, new groups were formed and other groups went extinct. A new group was typically formed via (i) two or more individuals dispersing from an established group to a new area and attracting others to that area through interaction with established groups or through calling behaviour to attract potential mates or (ii) via floaters joining together. Floaters are individuals that have left their group, but have not managed to disperse into a new group, and hence are ‘floating’ in the space between established groups [38].

Each babbler group was observed once per week for 3–4 h per observation session. Life-history data, including all breeding activity, dispersal and roving events were recorded. During each observation session from 2007 to 2015, GPS location points were collected from the centre of the foraging group every 15 min using handheld GPS devices. Home ranges were established on an annual basis for each group, with 16 groups over the 8 years of GPS data collection meeting the minimum criteria of 300 GPS points to allow a territory size calculation (*n* = 60 annual territory sizes calculated, 30 within-group territory sizes paired between consecutive years). Territory size estimates reached an asymptote at just below 300 points once all group territory size estimates were considered—indicating 300 was likely a sufficient sample number to give a reliable estimate of territory size. Group territory sizes were calculated using the ‘adaptive sphere-of-influence local convex hull’ (a-LoCoH) methodology [39]. A-LoCoH was performed using a minimum of 300 location points for each group. Home range sizes were calculated from 95% density isopleths in ArcGIS 9.3.1 (ESRI, 2009).

Kidnapping was defined as the physical removal of a bird from its group while the parents and other group members were still present and providing care for it, distinguishing this behaviour from adoption, where individuals may be observed associating with orphaned individuals [30]. Kidnapping was a process that was resisted by the group members of the abductee, with intergroup fighting commonly observed prior to, during and after the kidnapping event [30]. The primary way that kidnapping occurs in pied babblers is as follows: adult members of the kidnapping group initiate an intergroup interaction with a neighbouring group. One member of the kidnapping group moves behind the defending group to locate their young (who do not participate in intergroup interactions), while the rest of the kidnapping group's members continue to invest in the intergroup interaction. Using a food item and a specific ‘feed vocal’ (as defined in [40] as a call given when adults are delivering food to young, which commonly results in young pursuing the calling adult), the kidnapping adult leads the young out of their natal territory and into the kidnapper's own territory.

Kidnapping intergroup young that are still dependent on adults for food may make kidnapping more successful: our previous experimental research has revealed that dependent young do not show kin recognition behaviours and instead will respond to the call of any adult, with kin recognition gradually developing as they age [41].

### (a) Analysis

Our analysis focused on the factors that may (i) affect group size and (ii) promote kidnapping behaviour or acceptance of rovers. First, we looked at the effect of chick recruitment to nutritional independence (three months post-fledging) on territory size, by comparing the change in territory size between the year that a group failed to raise any young with the year when they did raise young (consecutive years) using a paired *t*-test. We conducted this analysis for all groups that successfully raised young during *both* years for comparison, to minimize the role of annual environmental influences behind observed territory size changes. To determine the relationship between group size and territory size, we conducted a linear mixed model (LMM) analysis with annual territory size as the response term and adult group size (mean number of adults present in the group annually) as a predictor term. Group identity was included as a random term to account for the potential influence of repeated measures.

To determine the relationship between group size and annual probability of extinction, we conducted a generalized linear mixed model (GLMM) analysis with a binomial distribution and a logit link function (where 0 = extant, 1 = extinct). A group was defined as extinct when the group was not detected at the study site at the start of the next breeding season after one month (eight visits) of searching the entire territory and surrounds and when some individuals from the former group were present in other groups that they had dispersed into (this differentiates group extinction from potential dispersal of the group to a new area outside of our study site detection zone; note that it also creates some differences to the theoretical kidnapping model, see §5).

We considered the following terms as potential predictors of group extinction: adult group size at the start of the breeding season, the occurrence of drought during the breeding season (coded as yes or no, where drought is defined as rainfall lower than 75% of the long-term average; see [42] for more details of drought at our study site), annual rainfall (mm), relative group size (defined as the size of the focal group/the average size of all groups in territories neighbouring the focal group) and number of breeding attempts during the breeding season and chick recruitment (the total number of young raised to nutritional independence that breeding season). Group identity was included as a random term in the analysis. The sample size included 269 group-year observations of 61 groups over 16 years.

To determine which groups were more likely to invest in kidnapping behaviour, we conducted a GLMM analysis with a binomial distribution and a logit link function where 0 = did not kidnap, 1 = kidnapped young. We included the following terms as potential predictors: drought (Y/N), annual rainfall (mm), adult group size, relative group size, chick recruitment and number of breeding attempts during each breeding season. Group identity was included as a random term in the analysis. The sample size included 269 group-year observations of 61 groups over 16 years.

During our daily observations, we recorded all cases of rovers approaching a group to within 10 m. Extragroup birds approaching groups is an atypical behaviour, as intergroup interactions normally occur as whole-group events where individuals engage in vocal and/or physical displays on their territory borders [10,30]. Rovers typically approach other groups when they are about to disperse from their natal group or when they are floating in the population [38]. Upon approach, rovers may either be chased away by the group or accepted by the group. Acceptance typically takes the form of non-aggressive approach, allopreening and vocalizing or foraging together. Once rovers join a group, they invest in helping behaviour similar to other group members [30]. To determine whether groups that did not successfully recruit young were more likely to accept rovers, we conducted a GLMM analysis with a binomial distribution and a logit link function (where 0 = did not accept rover into the group, 1 = accepted into group). Potential predictors of rover acceptance included adult group size at the start of the breeding season, the occurrence of drought during the breeding season, annual rainfall (mm), relative group size, number of breeding attempts and chick recruitment. Group identity was included as a random term in the analysis. The sample size included 265 group-year observations of 61 groups made over 16 years. This sample size is slightly different to that for the kidnapping data due to the exclusion of ambiguous rover records in four cases.

**Figure 1.**
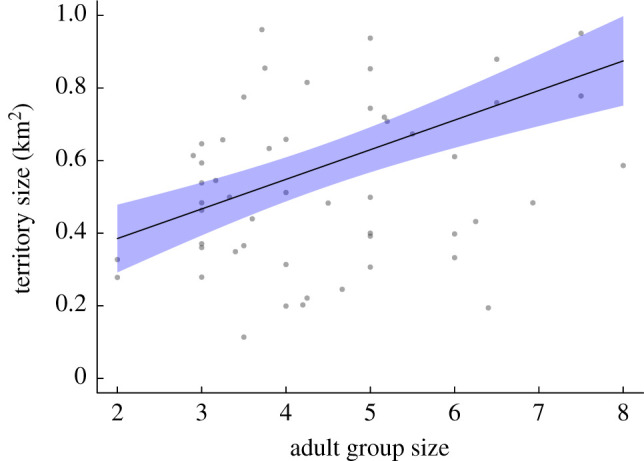
The relationship between adult group size and territory size. The line of best fit is generated from the output of the LMM: shaded areas represent s.e.; grey dots are raw data. (Online version in colour.)

**Figure 2.**
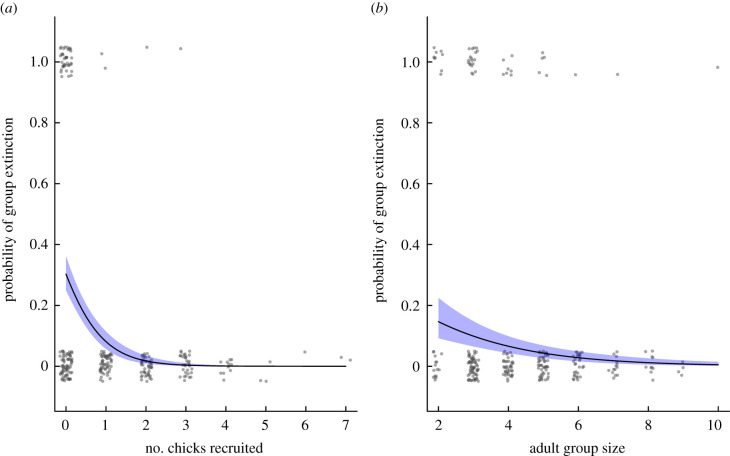
The relationship between the probability of group extinction and (*a*) number of chicks recruited during the breeding season and (*b*) adult group size. Curves are generated from the output of the model presented in table 1: shaded areas represent s.e.; grey dots are raw data. (Online version in colour.)

**Figure 3.**
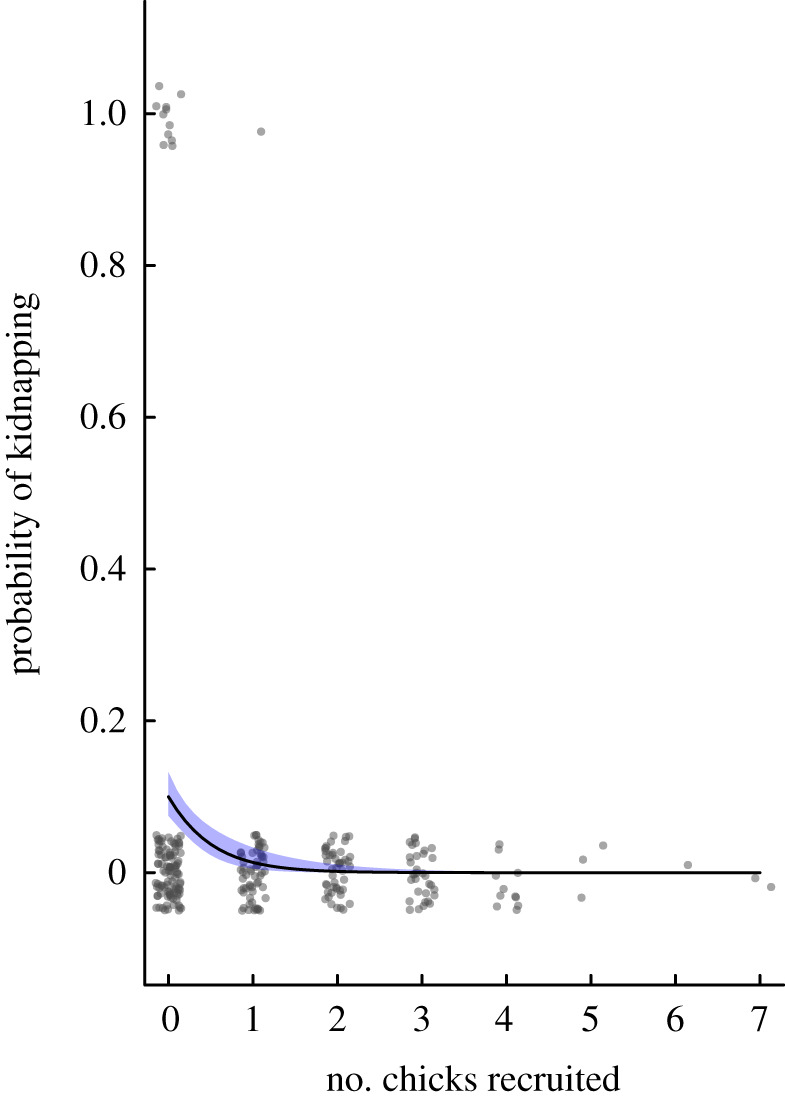
The relationship between the probability of kidnapping behaviour and chick recruitment within the focal group during each breeding season. The curve of best fit is generated from the output of the model presented in table 2: the shaded areas represent s.e.; grey dots represent raw data. (Online version in colour.)

**Figure 4.**
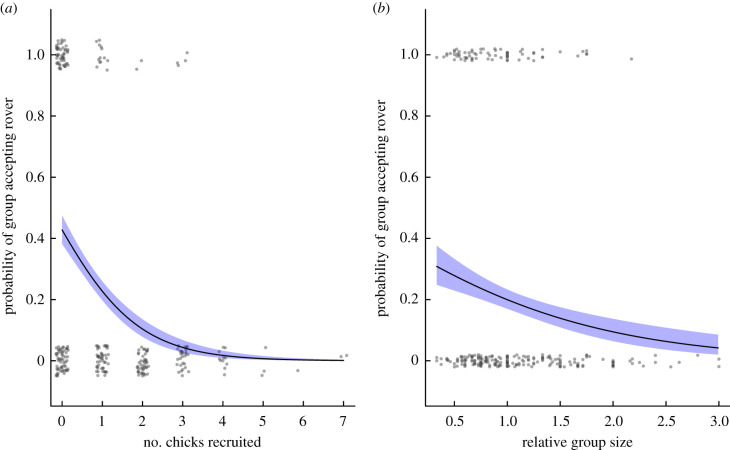
The relationship between the probability of a group accepting a rover and (*a*) the number of chicks recruited during the breeding season and (*b*) relative group size. Curves are generated from the output of the model presented in table 3: shaded areas represent s.e.; grey dots represent raw data. (Online version in colour.)

We used AICc (Akaike's Information Criteron corrected for small sample sizes) model selection to determine a top model set. Where two models shared very similar AICc values, but one model had a greater number of predictors than the other, we used the simpler model as per Harrison *et al.* [43]. We considered terms were important predictors of the data if their confidence intervals did not intersect zero. Correlated terms were not included in the same models together if the VIF (variance inflation factor) > 3. All analyses were conducted in IBM SPSS statistics v. 27.0.1.0. Figures 1–4 were drawn in R v. 4.0.5 (R Core Team, 2021) using minimal models identified through AICc model selection.

**Table 1.** Model selection of the terms influencing the likelihood of a group going extinct per annum. Group identity was included as a random term. Analysis is conducted on 269 group-years for 61 babbler groups for the period spanning 2003–2019. The top model is in italics.

parameter		AICc	ΔAICc
*chick recruitment + adult group size*		*169.91*	*0*
chick recruitment		170.48	0.57
chick recruitment + relative group size		173.10	3.19
no. of breeding attempts		193.34	23.43
adult group size + no. of breeding attempts		193.96	24.05
drought Y/N + adult group size		208.44	38.53
adult group size		208.91	39.00
relative group size		215.69	45.78
null		219.16	49.25
drought Y/N		220.24	50.33
annual rainfall		220.66	50.75
**effect sizes for top model**			
**parameter**	**estimate**	**s.e.**	**95% CI**
intercept	2.03	0.93	0.23, 3.88
adult group size	0.38	0.20	0.07, 3.70
chick recruitment	1.55	0.49	0.59, 2.50

**Table 2.** Model selection of the terms influencing the occurrence of kidnapping (0 = no kidnapping by focal group, 1 = focal group invested in kidnapping behaviour). Group identity was included as a random term. Analysis is based on 269 group-years of data from 61 groups. The top model is in italics.

parameter		AICc	ΔAICc
*chick recruitment + relative group size*		*69.40*	*0*
chick recruitment		70.82	1.42
chick recruitment + adult group size		71.26	1.86
no. of breeding attempts		73.35	3.95
relative group size + no. of breeding attempts		73.77	4.37
relative group size		76.86	7.46
null		79.36	9.96
adult group size		79.67	10.37
annual rainfall		80.14	10.74
drought Y/N		80.93	11.53
**effect sizes for top model**			
**parameter**	**estimate**	**s.e.**	**95% CI**
intercept	1.04	0.79	−0.51, 2.59
chick recruitment	1.79	0.80	0.23, 3.34
relative group size	1.59	1.19	−0.74, 3.92

**Table 3.** Model selection of the terms influencing the acceptance of rovers (where 0 = attempted immigration event repelled by group, 1 = attempted immigration event not repelled). Group identity was included as a random term. Analysis is based on 336 roving events recorded at 61 different groups during 265 group-years. The top model is in italics.

parameter		AICc	ΔAICc
*chick recruitment + relative group size*		*241.94*	*0*
chick recruitment + adult group size		244.88	2.94
chick recruitment		248.38	6.44
relative group size		281.85	39.93
adult group size		285.79	43.85
no. of breeding attempts		291.86	49.92
annual rainfall		293.09	51.15
null		293.26	51.32
drought (Y/N)		295.18	53.21
**effect sizes for top model**			
**parameter**	**estimate**	**s.e.**	**95% CI**
intercept	−0.87	0.39	−1.65, −0.11
chick recruitment	0.98	0.24	0.50, 1.46
relative group size	1.02	0.38	0.27, 1.80

## 3. Results

### (a) The relationship between territory size and group size

There was large variation in annual territory size within the population, ranging between 0.11 and 1.84 km^2^ (mean 0.61 ± 0.04 km^2^), with larger groups tending to occupy larger territories (LMM: *F* = 0.81, *p* = 0.008, figure 1). Groups that failed to recruit young had a significantly smaller territory size compared to years when they did successfully recruit young (paired *t*-test, *t*_8_ = –3.15, *p* = 0.016). On average, group territory size declined by 26.8% in years when there was no successful breeding compared to successful breeding years. By contrast, groups that successfully recruited young in both years showed no significant inter-annual variation in territory size (paired *t*-test, *t*_24_ = 1.39, *p* = 0.18).

### (b) Group extinction

There were 44 group extinction events over the 269 group-years of the study period. Chick recruitment was an important predictor of group extinction, with groups that failed to raise any young during a breeding season more likely to go extinct before the next breeding season (table 1 and figure 2*a*). From 697 breeding attempts in 61 groups, there were 110 group-years (of 269 group-years) where groups failed to raise any young over the entire duration of a breeding season. Of these cases, 40 groups (37.2%) went extinct before the next breeding season. By contrast, of 159 group-years in which groups successfully raised young to nutritional independence, only four groups (2.5%) went extinct before the next breeding season. Group size was also an important predictor of extinction, with small groups more likely to go extinct than large groups (table 1 and figure 2*b*).

### (c) Are groups that fail to raise chicks more likely to kidnap young?

Groups that did not recruit any chicks during a breeding season were more likely to kidnap young from other groups (table 2 and figure 3). Since it is also relevant to test the model prediction that small groups should be more likely to kidnap chicks, we included a model where group size is an explanatory factor, either alone (leading to ΔAIC_c_ = 79.67) or together with the failure to recruit chicks (leading to ΔAIC_c_ = 71.26, which is within the range of models of moderate support). There is therefore stronger evidence for failure of chick recruitment as the proximate trigger of kidnapping, than for adult group size *per se*.

Only one group that successfully raised their own chick kidnapped a chick from another group. This group raised one chick of their own after three failed breeding attempts. The average age of kidnapped individuals was 47.9 ± 6.1 days post-hatching. At this age, young are not nutritionally independent and continue to be provisioned by their kidnappers [30,36]. Kidnapping groups successfully retained kidnapped young in 40% of cases; in the remaining cases, natal groups successfully retrieved their young. The average duration of kidnapping for unsuccessful events was 2.8 d. All kidnapping events occurred in the latter half of the breeding season (mean = 149.6 ± 14.2 d since the first breeding attempt of the season) and only after groups had attempted to raise their own young, with an average of 2.3 failed breeding attempts per group prior to kidnapping behaviour.

### (d) Are groups that fail to raise chicks more likely to accept rovers?

If the recruitment of individuals is key to group longevity, then we would expect to see individuals in groups that failed to breed undergoing strategies to increase or maintain group size, such as acceptance of rovers into the group. We found that groups that failed to raise any young in a breeding season were more likely to accept rovers into their groups than groups that did raise young (figure 4*a* and table 3). Relative group size also affected the probability of acceptance of rovers into the group: groups that were smaller than neighbouring groups were more likely to accept rovers (figure 4*b*).

## 4. Model

Here, we provide a theoretical analysis that considers how kidnapping may impact long-term genetic contributions of a given group member to future generations, if the group kidnaps or does not kidnap, given an opportunity to do so. The model is based on the logic that the benefits of group augmentation due to kidnapping must outweigh the cost of inclusive fitness through reproductive competition. We ask how large the benefit needs to be (in terms of reduction in the probability of a group going extinct), for groups of different sizes and relatedness values, for kidnapping to pay.

Since kidnapping involves collective behaviour, we consider kidnapping to be adaptive if a randomly chosen group member can improve its expected genetic representation in the long term when kidnapping occurs over the expectation when it does not occur. If the expected representation improves for a randomly chosen member, then we assume that group members as a whole have an incentive to participate in the kidnapping event. Obviously, this is a simplification since real groups may have class structure where certain individuals have more power in the decision-making, and this may covary with their reproductive value, age or sex; however, in the absence of any data other than those represented in figure 2 regarding how a decision is made, we consider the average improvement of a randomly chosen focal individual the best proxy for a first attempt to understand the relevant issues theoretically.

To provide the simplest setting that allows us to contrast the benefits and costs of kidnapping, we consider the superior performance of groups that grow in size (figure 2), together with kidnapping of non-kin having a diluting effect on local relatedness. Since there is no reason to expect that kidnapped individuals are excluded from ever becoming reproductives, this implies that kidnapping leads to more competition from non-relatives for genetic representation in future generations, should the kidnapped individual and the randomly chosen focal individual be of the same sex.

Consider that a group that is currently of size *N* has the probability *p*(*N*) of going extinct, i.e. having no living descendants to contribute to the global population in the long term. We assume that smaller groups have a higher risk of extinction, and this means that *p*(*N*) is a declining function of *N*. The fact that kidnapped individuals can act as helpers (after an initial period during which they need to be fed) is implicit in the *p*(*N*) decreasing with *N*. Note that it is biologically possible that the effort of rearing the kidnappee is so large that, as a net effect, *p*(*N* + 1) no longer represents an improvement over *p*(*N*). Our model aims to predict how large the improvement needs to be, i.e. how strong the net benefit (‘net’ reflecting the fact that persistence improvement must also implicitly include, and overcome, the detrimental effects of the initial rearing effort) has to be to overcome another cost, which is the competition to be an ancestor of future individuals. The latter cost is also expressable as dilution of relatedness of the average individual who contributes to a specific future individual.

From the perspective of an existing group member, the fitness consequences of adopting an unrelated group member depend on the latter's sex. Juveniles of this species (like most birds) are sexually indistinguishable; therefore, the decision to kidnap has to be made without knowing this in advance. Alternatively, it is possible that adults can assess the sex of juveniles, but even in this case, the nature of collective decision-making requires that the group as a whole, consisting of both males and females, needs to on average benefit from the decision. This represents an alternative justification for our approach—though this argument ceases to be valid if individuals of one sex have more impact on collective decision-making than others; in the absence of any evidence to support such a power asymmetry, we do not include it in the model. We assume that sex ratio is 1 : 1 among the kidnappers and abductees alike. Therefore, the focal existing group member and the abductee are of the same sex with probability ½ and of a different sex with probability ½. Further, we assume that a focal group member is related to current group members with relatedness *r*_,_ while the relatedness to the juvenile that represents an opportunity to kidnap is zero.

If kidnapping does not occur, there will be future descendants of the group with probability 1–*p*(*N*); we compute the fitness gain via a given descendant long in the future for a randomly chosen current group member. If there is no kidnapping, we simply assume that any one individual in the current group can be the one who produces the offspring who is part of the lineage leading to the focal descendant. We assume inbreeding avoidance (common in cooperative breeders and observed in pied babblers [37]) and therefore assume that either the focal individual itself is the ancestor and it mates with an unrelated group member or that the focal group member is not involved in the breeding event that produces the offspring that founds the lineage in question. In the latter case, we consider *r* as the average of two relatedness values: that of the male parent to the focal individual and that of the female parent to the focal individual. We expect this mean of two *r* samples not to differ, on average, from the overall mean *r* in the local group (we are comfortable with this assumption as our results show that the value of *r* proves to have remarkably little effect on the results; note that many situations of inbreeding avoidance imply that one of the parents will be picked among non-relatives or distant relatives of the focal individual and another among closer relatives).

Consider the ‘no kidnapping’ case first. The focal offspring is assumed to have only one of its two parents with relatedness to the focal individual, reflecting our assumption of inbreeding avoidance. This is therefore also the only parentage that matters for the fitness calculations (the other parent's fitness does not matter for our proxy for fitness). The probability that this parent is the focal individual itself is 1/(*N*/2), where the factor 2 relates to the fact that there are two sexes: a total of *N*/2 individuals compete locally to be ancestors of the current individual among that sex.

If the focal individual is not the ancestor (probability 1–1/(*N*/2)), then we consider *r* as the fitness gain (which may arise either via males or females as explained above), and we have a proxy for fitness $(1-p(N))\left(\frac{1}{N/2}+r\left(1-\frac{1}{N/2}\right)\right)$.

If the kidnapping does occur, the extinction probability decreases from *p*(*N*) to *p*(*N* + 1). Simultaneously, the probability 1/(*N*/2) that the focal individual is directly involved in producing the offspring changes to 1/((N/2)+1), if the kidnappee is a same-sex competitor. This change occurs with probability ½, and it is also associated with a dilution of *r* in the group as a whole, as detailed below. With the complementary probability ½, the kidnappee is of the opposite sex, and the term 1/(*N*/2) stays unchanged, but the dilution of *r* still happens.

The dilution changes *r* to a new value that we denote *r*′. Here *r*′ is the mean of two values: the previous relatedness *r*, which was the mean that applies for *N*–1 individuals (the –1 arises because we use *r* to denote relatedness to others excluding self), and relatedness zero for one new individual. The weighted mean is *r*′ = (*N*–1)*r*/*N*.

As a whole, the inclusive fitness term, from the focal member's point of view, changes from $\left(\frac{1}{N/2}+r\left(1-\frac{1}{N/2}\right)\right)\;$ to $\frac{1}{2}\;\left(\frac{1}{\frac{N}{2}+1}+r^{\prime }\left(1-\frac{1}{\frac{N}{2}+1}\right)\right)+\frac{1}{2}\;\left(\frac{1}{N/2}+r^{\prime }\left(1-\frac{1}{N/2}\right)\right)$, where *r*′ = (*N*–1)*r*/*N*. The overall fitness proxy for the case where kidnapping occurs is obtained by multiplying by (1−p(N+1)) and simplifying, yielding the left-hand side of the inequality that decides whether kidnapping is adaptive

4.1a $$(1-p(N+1))\frac{N^{3}r+N^{2}\;(2-r)+2N\;(1-r)+2r}{N^{2}(2+N)}>(1-p(N))\left(\frac{1}{N/2}+r\left(1-\frac{1}{N/2}\right)\right)$$

This can be simplified to

4.1b $$\frac{1-p(N+1)}{1-p(N)}>\frac{N\;(N+2)(2+r\;(N-2))}{N^{3}r+N^{2}\;(2-r)+2N\;(1-r)+2r}.$$

**Figure 5.**
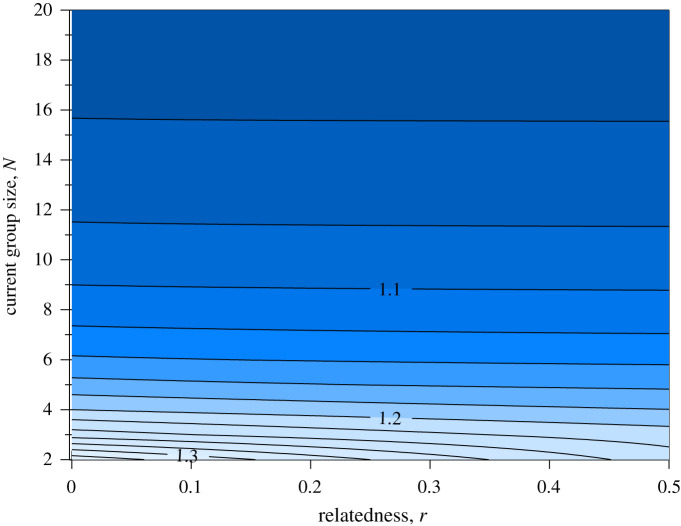
Values of the right-hand side of equation (4.1*b*) as a function of relatedness and group size. If the survival odds improvement is of at least the indicated magnitude (e.g. 1.2 = 20% improvement) when group size increases by one individual, then the benefits of kidnapping exceed the costs in the present situation. (Online version in colour.)

The left-hand side of this equation represents the survival odds improvement of the entire group when kidnapping one new group member. The right-hand side gives the minimal requirement for this to happen. This threshold is higher for small *N* and depends only very mildly on *r*. For realistic ranges of relatedness *r* and group size *N*, the threshold values are somewhat above 1 (figure 5), where a value of e.g. 1.2 means that kidnapping is adaptive from the perspective of a randomly chosen group member if the survival of the group improves by at least 20% when one young is added to the group; it is not adaptive if the survival improvement is lower.

If one relied on ‘all else being equal’ predictions, one might predict that kidnapping pays in large groups (darkest blue in figure 5). However, it is highly unlikely that all else is equal across different-sized groups; the impact of additional (helpful) individuals on survival odds is often larger in small than in large groups.

Ultimately, the values of group-level survival odds, and how they change with the addition of one group member, are an empirical question. The data in figure 2 suggest that group extinction diminishes rapidly when moving from no recruitment to recruitment of one chick, and the curve is relatively flat thereafter (low extinction probability). The predicted extinction probability is 0.303 when no chick was recruited, dropping to 0.081 when one chick was recruited. The corresponding survival values are 0.697 and 0.919, i.e. the improvement estimate is 1.319, which is above the threshold value that is close to 1.2 in the range for the typical group sizes in our population (most groups are between *N* = 3 and *N* = 6, figure 1's *x*-axis); note that this holds over all realistic values of *r* between 0 and 0.5 (figure 5). The odds improvement of 1.3 is, interestingly, larger than the largest value we find in figure 2*b*, where the fitted extinction probabilities (as a function of group size) have pooled both successful and unsuccessful groups with respect to chicks recruited. In figure 2*b*, the steepest predicted survival change is in smallest groups: improving group size from two to three yields a drop from 0.146 to 0.099 in estimated extinction probabilities, i.e. survival improvement from 0.854 to 0.901, yielding 1.055 as the improvement; this value, based on figure 5, is not a sufficiently large benefit for kidnapping to pay *on average* for a group member for the range of group sizes that babblers form.

Based on these numbers, we can conclude the following. In an approach that pools all data for a given group size (figure 2*b*), extinction risk is not sufficiently lowered by the addition of a group member for kidnapping to pay on average. However, the estimates in figure 2*b* do not distinguish between groups that failed to produce young and those that did. The failed groups are those that, for any given *N*, are at higher risk. Focusing on this subset of groups (left end of figure 2*a*) shows there to be a subset of conditions where an additional group member makes a sufficient difference to make the average group member benefit, as a net effect, from kidnapping. If zero chicks were recruited, the data-driven estimate of extinctions reaches values where the model predicts kidnapping to be adaptive regardless of the precise values of *r* and *N*. Kidnapping events in reality are very strongly associated with zero recruitment of own chicks (figure 3), and we can conclude that the model predicts babblers to be as a whole in an interesting borderline situation: kidnapping does not pay uniformly across all situations but can pay facultatively in situations where own reproduction has failed.

## 5. Discussion

We found that group size, and particularly the failure to have produced young that recruit into the group, was an important determinant of extinction risk, with small groups suffering a higher annual extinction probability. Such critical group size effects have been described in several cooperative species [14,15] and highlight the importance of group size to group longevity. Under such circumstances, kidnapping and increased acceptance of rovers may be considered adaptive responses to prevent groups declining to a size where they face a high probability of extinction. Our data supported this: kidnapping behaviour in pied babblers was highly predictable and appeared to be driven by unsuccessful breeding attempts. Our modelling of the required improvements of group survival reveal that the babbler groups are not uniformly finding themselves in circumstances where kidnapping pays; however, those groups that have failed to raise their own young during the present breeding season are, on average, in a situation where the benefits of kidnapping may exceed the costs. Empirically, there is a good match to this prediction: with the exception of one event where kidnapping and successful production of own young co-occurred, kidnapping was always preceded by the group failing to have produced any intragroup young.

The fact that successful intragroup breeding makes intergroup kidnapping behaviour less likely suggests that kidnapping involves effort that makes it a last resort option. It generally only occurs at the end of the breeding season after groups had tried to raise their own young. Groups with no breeding success face a high possibility of decline in territory size and group size before the next breeding season due to the probable stochastic events of predation, dispersal and death of current group members [44]. Kidnapping young may therefore be a way to maintain group size that is only adaptive when other alternatives fail. Its facultative nature predicts limited occurrence: groups do not routinely exchange offspring. This latter alternative, although hypothetical, would differ from our kidnapping scenario as it could be hypothesized to reflect intergroup cooperation: in this alternative view, dispersal is a social trait that alleviates kin competition in the natal group, and neighbouring groups could help each other to achieve dispersal by actively leading juveniles to their new homes. The clear indications of intergroup conflict during a kidnapping event (see §2), as well as the rarity of kidnapping, speak against this alternative.

Kidnapping events involved the taking of young individuals only, and this pattern has been observed in most recorded cases of kidnapping in non-human animals [23–26,28]. This raises the question of why kidnappers prefer to take dependent young. The answer to this may lie in imperfect kin recognition systems: if young learn to recognize individuals based on who fed them during their developmental period [22,45,46], then provisioning behaviour by kidnappers could suffice to establish a relationship between kidnapper and abductee similar to the bonds observed in kin relationships [46]. Abductees may then become helpers in their new group and provide future benefits for their kidnappers, without incurring costs via intragroup reproductive competition, as has previously been recorded for this species [47,48]. This future helping behaviour has been observed in both pied babblers and white-winged choughs [25], suggesting that kidnapping behaviour can result in multiple benefits and may indeed be an adaptive, facultative response when intragroup breeding attempts fail.

If kidnapping behaviour can be explained as a strategy to maintain or increase group size when breeding attempts fail, then we would expect to see other behaviours that help to maintain or increase group size being displayed as well [19,49], and indeed we did observe this: groups whose breeding attempts failed were significantly more likely to accept rovers into their group. This could be explained by (i) these groups being less able to defend against rovers looking for a group to join or (ii) these groups being more willing to accept rovers into their group. We suggest that (ii) is the most likely explanation because there was no difference between groups that failed to breed or not in terms of the number of times rovers visited the groups. Instead, there was a difference in the likelihood of rovers being accepted into the group. Accepting rovers into a group is a risk: these adults may act as reproductive and foraging competitors [47,50–52]. However, our analysis revealed that the cost of not gaining additional group members can be group extinction. Therefore groups where breeding attempts have failed to recruit any young may be more likely to accept rovers because the benefits of additional group members outweigh the potential cost of group extinction or reproductive competition, fitting in well with the theoretical predictions of Keller & Reeve [49].

If a group becomes extinct but individuals from that group can disperse to other groups, then is group extinction costly to group members? Our model approximated the fitness of members of groups that go extinct as zero, on the grounds that success of its members, if forced to disband, is likely to be very low. While we have observed some individuals from extinct groups dispersing to other groups, these dispersals are primarily into subordinate, non-breeding positions or into small groups that often disband without reproducing. We therefore consider group extinction a significant cost to former members of the extinct group, since floaters lose condition [38], dispersal into large groups is unlikely because large groups are less likely to accept rovers (as seen in our analysis above) and small, newly formed groups can be ephemeral and often disband without reproducing. Our decision to treat extinction with zero fitness in our model means that we may have overestimated its severity, which would then mean overestimating the adaptive value of kidnapping. However, we also made other approximations that are likely to cause deviations in the opposite direction: we assumed that incoming juveniles count as much as any other group member as competitors for reproductive opportunities. If juveniles rarely succeed in becoming a reproductive competitor to the kidnapper, this may again enlarge the zone in which kidnapping is adaptive (then again, if juveniles fail because they die, their positive potential effects as helpers also disappear from that point on, making kidnapping a poorer investment). Many additional factors that we omitted (e.g. the dynamic nature of the territory size that can be defended) may tilt the balance from figure 5 somewhat in either direction. As a whole, we believe our model is a first step towards understanding why kidnapping may be facultative, not a routine expectation in group-living species.

Relative group size may be a better predictor of intergroup behaviour than actual group size in group-living species, since the level of competition or threat posed by surrounding groups may depend on their size relative to that of the focal group. An increasing number of studies in group-living species are assessing relative group size [33,53], and our data support the importance of this parameter: individuals were more likely to accept rovers into their group when focal group size was low relative to surrounding groups. This suggests the ability to (i) assess the size of neighbouring groups relative to one's own group and (ii) respond by investing in behaviours (e.g. tolerance of extragroup individuals) that increase group size when relative group size is low. We suggest that greater inclusion of relative group size into studies on intergroup interactions may allow a broader, perhaps more comprehensive overview of factors affecting intragroup behaviour.

An alternative strategy to increase group size relative to one's neighbours could be infanticide. Infanticide is a behaviour observed during intergroup interactions in a number of species (reviewed in [54]), but was never observed in pied babblers during intergroup interactions (occasional injury was observed, but not offspring mortality). There may be several reasons for this: (i) babblers may not be physically capable of committing infanticide or (ii) the benefits of kidnapping young outweigh the benefits of infanticide. In white-winged choughs, kidnapped young became helpers in their new group [25], and the same was observed in pied babblers: kidnapped young were observed helping to raise the young of their kidnappers, in some cases at multiple broods for several years. Kidnappers were not aggressive towards kidnapped young, but instead displayed alloparental care. This may enhance bonding between the kidnapper and the kidnapped young, similar to that observed in slave-making ants [22].

Our research has confirmed that group size can be an important factor affecting group extinction. Therefore, when common behaviours that increase group size (such as recruitment of young) fail, group members may adopt alternative strategies to increase group size. Although these alternative strategies are less common and thus in general less well documented in the existing literature, our long-term data have revealed that both acceptance of rovers and kidnapping behaviour are predictable and strongly related to the breeding success of the focal group, thus confirming predictions of the group augmentation hypothesis [19]. Importantly, both these behaviours are intergroup behaviours: acceptance of adults and kidnapping of intergroup young require interaction with other groups. Kidnapping in particular can involve highly aggressive interaction between the kidnapping and natal group. Thus, these two behaviours illustrate the importance of intergroup interactions on intragroup behaviour: either voluntarily in the case of rovers or involuntarily in the case of kidnapping. We thus suggest that the data presented here add to the growing body of evidence [55] suggesting that intergroup interaction is a vital factor to consider in order to fully understand the dynamics of group-living behaviour.

## Data Availability

The data are provided in electronic supplementary material [56].
